# Maternal migraine and the risk of psychiatric disorders in offspring: a population-based cohort study

**DOI:** 10.1017/S2045796021000421

**Published:** 2021-07-15

**Authors:** H. Wang, H. He, M. Miao, Y. Yu, H. Liu, J. Zhang, F. Li, J. Li

**Affiliations:** 1 MOE-Shanghai Key Laboratory of Children's Environmental Health, Xin Hua Hospital Affiliated to Shanghai Jiao Tong University School of Medicine, Shanghai, China; 2 Department of Clinical Medicine-Department of Clinical Epidemiology, Aarhus University Hospital, Aarhus, Denmark; 3 Department of Developmental and Behavioral Pediatric & Child Primary Care, Xin Hua Hospital Affiliated to Shanghai Jiao Tong University School of Medicine, Shanghai, China; 4 NHC Key Lab. of Reproduction Regulation (Shanghai Institute of Planned Parenthood Research), Fudan University, Shanghai, China; 5 Department of Biostatistics, School of Public Health, and The Key Laboratory of Public Health Safety of Ministry of Education, Fudan University, Shanghai, China; 6 School of Public Health/Medical Informatics Center, Peking University, Beijing, China

**Keywords:** cohort study, migraine, psychiatric disorders, register-based research

## Abstract

**Aims:**

Maternal migraine may contribute to mental heath problems in offspring but empirical evidence has been available only for bipolar disorders. Our objective was to examine the association between maternal migraine and the risk of any and specific psychiatric disorders in offspring.

**Methods:**

This population-based cohort study used individual-level linked Danish national health registers. Participants were all live-born singletons in Denmark during 1978–2012 (*n* = 2 069 785). Follow-up began at birth and continued until the onset of a psychiatric disorder, death, emigration or 31 December 2016, whichever came first. Cox proportional hazards model was employed to calculate the hazard ratios (HRs) of psychiatric disorders.

**Results:**

Maternal migraine was associated with a 26% increased risk of any psychiatric disorders in offspring [HR, 1.26; 95% confidence interval (CI), 1.22–1.30]. Increased rates of psychiatric disorders were seen in all age groups from childhood to early adulthood. Increased rates were also observed for most of the specific psychiatric disorders, in particular, mood disorders (HR, 1.53; 95% CI, 1.39–1.67), neurotic, stress-related and somatoform disorders (HR, 1.44; 95% CI, 1.37–1.52) and specific personality disorders (HR, 1.47; 95% CI, 1.27–1.70), but not for intellectual disability (HR, 0.84; 95% CI, 0.71–1.00) or eating disorders (HR, 1.10; 95% CI, 0.93–1.29). The highest risk was seen in the offspring of mothers with migraine and comorbid psychiatric disorders (HR, 2.13; 95% CI, 1.99–2.28).

**Conclusions:**

Maternal migraine was associated with increased risks of a broad spectrum of psychiatric disorders in offspring. Given the high prevalence of migraine, our findings highlight the importance of better management of maternal migraine at childbearing ages for early prevention of psychiatric disorders in offspring.

## Introduction

Psychiatric disorders affect one in five people (Charlson *et al*., 2019). The aetiology of psychiatric disorders involves interaction of genetic, environment and lifestyle behaviours (Kraemer *et al*., 2001). Even though genetic components might be significant contributors to many psychiatric disease, increasing empirical evidence have shown that adverse early-life environment, starting in utero or even before, may increase the lifetime risk of mental health problems (Tegethoff *et al*., 2011). Research on prenatal origins of those diseases would provide important knowledge for developing more effective prevention strategies, which may open a new era of disease control for mental health problems (O'Donnell and Meaney, 2017).

Migraine is the most common chronic neurovascular disorder, ranking the second leading cause of years of life with disability (Vos *et al*., 2017; Dodick, 2018). Women are three times more likely to experience migraine than men, and are predominantly affected during their childbearing years (Burch *et al*., 2018). There is growing concern of the long-term mental health problems in the children born to mothers with migraine (Evans *et al*., 2005; Kaasbøll *et al*., 2012; Güngen *et al*., 2017). Children of mothers with migraine had more psychological and behavioural problems that were assessed through questionnaires in several previous studies (Evans *et al*., 2005; Kaasbøll *et al*., 2012; Güngen *et al*., 2017). It was suggested that maternal migraine may affect offspring psychiatric disorders via altered intrauterine environment in the central nervous system (Burch, 2020). If this hypothesis holds true, we should expect that maternal migraine would be associated with a higher risk of psychiatric disorders in offspring. Only one study examined maternal migraine and bipolar disorder in offspring (Sucksdorff *et al*., 2016). To our knowledge, no research has provided a comprehensive evaluation of the mental health outcomes of these children exposed to maternal migraine.

We hypothesised that maternal migraine could affect the fetal brain development and consequently mental health in offspring throughout the lifespan (Gandal *et al*., 2018). The aim of this study was to investigate the association of maternal migraine with any or specific psychiatric disorders in offspring, taking into account the timing of maternal migraine diagnosis and a number of other factors that may affect the association (McLaughlin *et al*., 2012; Skajaa *et al*., 2019).

## Methods

### Study population

We conducted a nationwide cohort study using data from the Danish national registers (Lynge *et al*., 2011; Mors *et al*., 2011; Wallach Kildemoes *et al*., 2011; Schmidt *et al*., 2015; Bliddal *et al*., 2018). In Denmark, all live births have a unique personal identification number that permits an accurate linkage of individual-level data. We identified all singleton live births from 1 January 1978 to 31 December 2012 (*n* =  2 105 712) from the Danish Medical Birth Registry (Bliddal *et al*., 2018) and excluded 461 children who had missing or extreme gestational age (<154 days or >315 days), 86 children without information on sex, 28 611 children with chromosomal abnormalities and 6769 children without links to their fathers. The final analysis included 2 069 785 children. We followed them from birth until the date of the first diagnoses of any psychiatric disorders, emigration, death or end of follow-up (31 December 2016), whichever came first. The Danish Data Protection Agency and the Danish Health Data Authority approved this study.

### Exposure

Information on maternal migraine before childbirth was obtained from the Danish National Patient Register (DNPR) and the Danish National Prescription Registry (Lynge *et al*., 2011; Wallach Kildemoes *et al*., 2011). The DNPR contains data on hospital admissions since 1977 and visits to outpatient clinics since 1995. Diagnoses for migraine are defined according to the *International Classification of Diseases, Eight Revision (ICD-8)* (1973 to 1993) code: 346 and the *International Classification of Diseases, Tenth Revision (ICD-10)* (1994 and onwards) code: G43 (Schmidt *et al*., 2015; Adelborg *et al*., 2018). A migraine case was also defined when the individual had at least two redeemed prescriptions for migraine-specific treatment (Anatomical Therapeutic Chemical (ATC) codes: N02CC (triptans) and N02CA (ergotamine)) (Skajaa *et al*., 2019). The index date of exposure was the date of the first diagnosis of migraine or the first date of the redeemed prescription, whichever came first.

### The outcome of interest

Information on psychiatric disorders was obtained from the DNPR and the Danish Psychiatric Central Research Register (Mors *et al*., 2011; Schmidt *et al*., 2015). Our primary outcome was the first diagnosis of a psychiatric disorder using *ICD* codes (*ICD*-8 codes from 1977 to 1993: 290–315; *ICD*-10 codes from 1994: F00-F99), which was further categorised into the following specific diagnostic groups: (1) schizophrenia and related disorders; (2) mood disorders; (3) neurotic, stress-related and somatoform disorders; (4) eating disorders; (5) specific personality disorders; (6) intellectual disability; (7) persasive developmental disorders; (8) behavioural and emotional disorders with onset usually occurring in childhood and adolescence (online Supplementary Table 1). When investigating the specific psychiatric disorders, we defined the date of onset as the first day of each specific psychiatric disorder diagnosis, irrespective of other previous psychiatric disorder diagnoses, if existed (Köhler-Forsberg *et al*., 2019).

### Covariates

Based on previous research (McGrath *et al*., 2014; Nilsson *et al*., 2017), the following variables were considered as potential confounders: sex of the child (male, female), calendar period of birth (a 5-year interval during 1978–2012), parity (1, 2, or ⩾3), maternal age at birth (⩽25, 26–30, 31–35, ⩾36 years), paternal age at birth (⩽25, 26–30, 31–35, ⩾36 years), maternal country of origin (Denmark, other countries), maternal education level (0–9, 10–14, ⩾15 years), maternal cohabitation status (yes, no), maternal psychiatric disorder history (yes, no), paternal psychiatric disorder history (yes, no) and maternal cardiovascular disease (*ICD*-8 codes: 390–459; *ICD*-10 codes: I00-I78) (yes, no). The information for maternal education and origin of country was obtained from the Danish Integrated Database for Longitudinal Labor Market Research (Petersson *et al*., 2011).

### Statistical analysis

We used Cox proportional hazards regression model to estimate the hazard ratio (HR) with 95% confidence interval (CI) for the association of maternal migraine with the risk of any or specific psychiatric disorders in offspring. Treating deaths from causes other than psychiatric disorders as competing events, we performed competing risk analysis to estimate cumulative incidences in exposed and unexposed offspring.

In model 1, we adjusted for sex and calendar year of birth. In model 2, we additionally adjusted for parity, parental age at birth, maternal education level, maternal income, maternal origin, maternal cohabitation, paternal migraine, parental psychiatric disorders before the childbirth and maternal cardiovascular disease. In addition, we tested whether the association between maternal migraine and the risk of psychiatric disorders in offspring varied by the sex of the child. We also separated the analyses according to offspring's attained age of psychiatric disorders. Age groups were set using cut-off points that captured potentially relevant development periods: 0–9 years (childhood), 10–18 years (adolescence) and > = 19 years (early adulthood) (Svahn *et al*., 2015).

As migraine is a chronic disease and there might be a lag in time for diagnosis (Weatherall, 2015), we took into consideration the timing of diagnosis (diagnosis before the childbirth, < = 2 years after the childbirth, 2–5 years after the childbirth, 5–10 years after the childbirth and >10 years after the childbirth) in separate analyses. We explored whether the risk for psychiatric disorders was different among children born to mothers with the following a priori defined mutually exclusive categories: no migraine and psychiatric disorders (referent), migraine, psychiatric disorders and migraine with psychiatric disorders (the joint effect).

We did several sensitivity analyses. First, in order to examine potential mediating effects of neonatal complications (Higgins *et al*., 2015), we performed the analyses after excluding children with preterm birth (<37 gestation weeks), low birth weight (<2500 g) and low Apgar score at 5 min (<7), to see whether the associations would be changed significantly, compared to those overall estimates. Second, due to the change on *ICD* codes (*ICD*-10 was adopted since 1994 in Denmark) and the migraine identification strategy (both outpatient diagnosis and prescription registry were available since 1995), we restricted the analysis to offspring born after 1996. Third, owing to the availability of data on maternal smoking (since 1991) and maternal pregnancy body mass index (since 2004), we restricted subanalyses to offspring born after 1991 and 2004, respectively. Fourth, to deal with the problems of missing values on covariates (i.e. maternal education level and cohabitation status), we consequently applied multiple imputation procedure by chained equations to impute ten replications to handle missing values of the confounders. Fifth, to capture the effect of migraine episodes during pregnancy, we additionally performed the analyses by further dividing exposure time window into two periods: prior to index prganncy and during index pregnancy. Lastly, to better evaluate the mediation effect, we conducted a mediation analysis to determine the proportion of the association between maternal migraine and psychiatric disorders in offspring that was mediated by the potential mediators (preterm birth, low birth weight and low Apgar score at 5 min). The mediators were assessed through multivariable logistic regression models of the outcome and the mediators; these results were then combined to estimate direct and indirect effects (via the mediators), adjusted for all the covariates as in model 2 (VanderWeele, 2016; Kim and VanderWeele, 2019). This mediation method assume that the covariates adjusted could adequately control exposure-outcome, mediator-outcome and exposure-mediator confounding (VanderWeele, 2016). The proportion mediated was calculated as log (natural indirect relationship)/log (total relationship). The mediation analyses were conducted using the PARAMED package in STATA. All statistical analyses were performed using STATA, version 15.1 (StataCorp).

## Results

Among 2 069 785 participants, 51 717 (2.5%) were born to mothers with migraine. The proportion of offspring born to mothers diagnosed with migraine increased over time (online Supplementary Fig. S1). Table 1 shows the baseline characteristics of children in the exposed and unexposed groups. Compared with unexposed offspring, exposed offspring were more likely to be born preterm, had low birth weight and older parents. Mothers of exposed offspring tended to have a higher level of education, a higher prevalence of comorbid psychiatric disorders or cardiovascular diseases.

**Table 1.** Characteristics of the study population born between 1978 and 2012 at birth according to maternal migraine status

	Maternal migraine status	
	Migraine	No migraine
Characteristics	*n* = 51 717	*n* = 1 800 517
Sex		
Boys	26 388 (51.0)	923 760 (51.3)
Girls	25 329 (49.0)	876 757 (48.7)
Preterm		
No	47 987 (92.8)	1 651 645 (91.7)
Yes	2974 (5.7)	81 886 (4.6)
Missing	756 (1.5)	66 991 (3.7)
Low birth weight		
No	48 816 (94.4)	1 705 162 (94.7)
Yes	2043 (3.9)	68 594 (3.8)
Missing	858 (1.7)	26 761 (1.5)
Apgar score at 5 minutes		
10	47 073 (91.0)	1 647 627 (91.5)
7–9	3442 (6.6)	108 597 (6.0)
0–6	386 (0.8)	15 063 (0.9)
Missing	816 (1.6)	29 230 (1.6)
Maternal age (years)		
⩽ 25	7383 (14.3)	484 928 (26.9)
26–30	18 421 (35.6)	684 346 (38.0)
31–35	17 723 (34.3)	463 094 (25.7)
> 36	8190 (15.8)	168 149 (9.4)
Paternal age (years)		
⩽ 25	3966 (7.7)	248 106 (13.8)
26–30	14 158 (27.3)	570 602 (31.7)
31–35	18 075 (35.0)	540 076 (30.0)
> 36	14 799 (28.6)	364 472 (20.2)
Missing	719 (1.4)	77 261 (4.3)
Maternal education level		
0–9	9817 (20.0)	490 319 (27.2)
10–14	24 153 (46.7)	786 075 (43.7)
⩾ 15	17 444 (33.7)	493 488 (27.4)
Missing	303 (0.6)	30 635 (1.7)
Maternal psychiatric disorders		
No	46 818 (90.5)	1 731 179 (96.1)
Yes	4899 (9.5)	69 338 (3.9)
Paternal psychiatric disorders		
No	49 017 (94.8)	1 738 192 (96.5)
Yes	2700 (5.2)	62 325 (3.5)
Maternal original		
Born in Denmark	47 603 (92.0)	1 603 856 (89.1)
Not born in Denmark	4103 (7.9)	192 006 (10.7)
Missing	11 (0.1)	4655 (0.2)
Maternal cohabitation status		
Yes	27 873 (53.9)	1 005 508 (55.8)
No	23 843 (46.1)	793 348 (44.1)
Missing	NA	1661 (0.1)
Maternal cardiovascular disease		
No	48 630 (94.0)	1 761 410 (97.8)
Yes	3087 (6.0)	39 107 (2.2)

Expresses as frequency (percentage); NA indicates less than three.

**Fig. 1.**
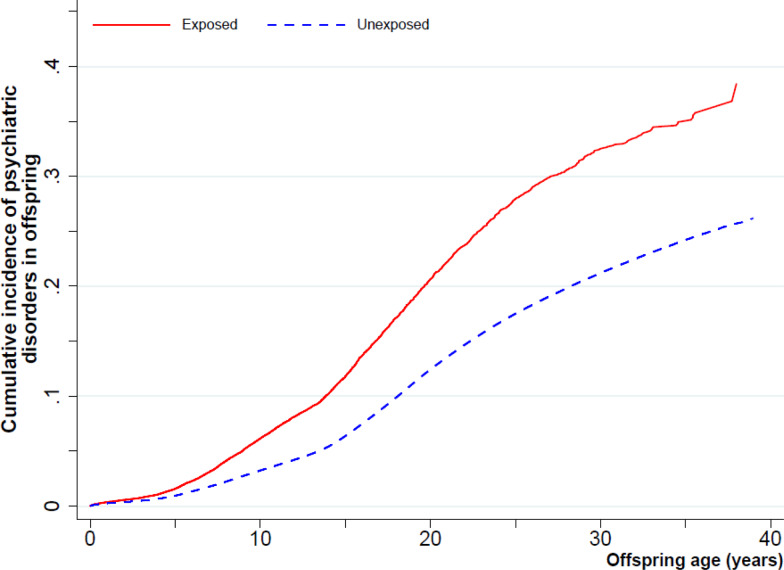
Cumulative incidence of overall psychiatric disorders among offspring exposed versus unexposed to maternal migraine.

The median follow-up time was 19 years (interquartile range: 11–27 years). 277 063 (13.4%) were diagnosed as having any psychiatric disorders. The cumulative incidence of psychiatric disorders was 38.4% (95% CI, 34.4–43.4) for the exposed offspring and 26.2% (95% CI, 26.0–26.4) for the unexposed offspring (Fig. 1). The crude incidence rates of any psychiatric disroders were 8.40 and 6.88 per 1000 person-years among offspring of mothers with migraine and without migraine, respectively. Compared with the unexposed offspring, exposed offspring had a 26% increased risk of any psychiatric disorders (HR,1.26; 95% CI, 1.22–1.30). There was a tendency that HRs increased with age, with the highest HR (1.34; 95% CI, 1.23–1.46) observed in the early adulthood (Table 2). Stratification by sex of the offspring did not indicate any significant differences (online Supplementary Table 2).

**Table 2.** Incidence rate and HRs of all psychiatric disorders in offspring according to maternal migraine status

	No. of offspring with psychiatric disorders	Rate per 1000	HR (95% CI)	HR (95% CI)
		person-years	Model 1	Model 2
Offspring psychiatric disorders				
Attained ages 0–39 years				
No maternal migraine	271 843	6.88	1 (Reference)	1 (Reference)
Maternal migraine	5099	8.40	1.32 (1.28–1.36)	1.26 (1.22–1.30)
Attained ages 0–9 years				
No maternal migraine	52 112	3.05	1 (Reference)	1 (Reference)
Maternal migraine	2216	5.41	1.26 (1.21–1.32)	1.22 (1.17–1.27)
Attained ages 10–18 years				
No maternal migraine	118 147	8.81	1 (Reference)	1 (Reference)
Maternal migraine	2309	13.97	1.32 (1.27–1.38)	1.26 (1.21–1.31)
Attained ages 19–39 years				
No maternal migraine	101 584	11.21	1 (Reference)	1 (Reference)
Maternal migraine	574	17.73	1.44 (1.32–1.56)	1.34 (1.23–1.46)

Model 1 adjusted for sex, birth year; Model 2 additionally adjusted for parity, maternal characteristic (age, education level, origin, cohabitation, cardiovascular diseases), paternal age, paternal migraine and parental psychiatric disorders before the childbirth.

Maternal migraine was associated with most of the specific psychiatric disorders in offspring, for example mood disorders (HR, 1.53; 95% CI, 1.39–1.67), neurotic, stress-related and somatoform disorders (HR, 1.44; 95% CI, 1.37–1.52) and specific personality disorders (HR, 1.47; 95% CI, 1.27–1.70). Maternal migraine was also associated with an increased risk of behavioural and emotional disorders with onset usually during childhood and adolescence (HR, 1.23; 95% CI, 1.17–1.28). Maternal migraine was not associated with intellectual disability (HR, 0.84; 95% CI, 0.71–1.00) or eating disorders (HR, 1.10; 95% CI, 0.93–1.29) in offspring (Table 3).

**Table 3.** Incidence rate and HR of specific psychiatric disorders in offspring according to maternal migraine status

	No of offspring with	Rate per 1000	HR (95% CI)	HR (95% CI)
	psychiatric disorders	person-years	Model 1	Model 2
Schizophrenia and related disorders				
No maternal migraine	19 251	0.84	1 (Reference)	1 (Reference)
Maternal migraine	166	0.89	1.30 (1.11–1.51)	1.21 (1.03–1.42)
Mood disorders				
No maternal migraine	45 608	2.02	1 (Reference)	1 (Reference)
Maternal migraine	485	2.61	1.61 (1.47–1.76)	1.53 (1.39–1.67)
Neurotic, stress-related and somatoform disorders				
No maternal migraine	98 606	3.16	1 (Reference)	1 (Reference)
Maternal migraine	1455	3.90	1.52 (1.44–1.60)	1.44 (1.37–1.52)
Eating disorders				
No maternal migraine	14 458	0.63	1 (Reference)	1 (Reference)
Maternal migraine	153	0.82	1.13 (0.96–1.33)	1.10 (0.93–1.29)
Specific personality disorders				
No maternal migraine	24 076	1.58	1 (Reference)	1 (Reference)
Maternal migraine	200	2.52	1.58 (1.38–1.82)	1.47 (1.27–1.70)
Intellectual disability				
No maternal migraine	9258	0.22	1 (Reference)	1 (Reference)
Maternal migraine	144	0.23	0.86 (0.73–1.02)	0.84 (0.71–1.00)
Pervasive developmental disorders				
No maternal migraine	25 375	0.61	1 (Reference)	1 (Reference)
Maternal migraine	865	1.37	1.15 (1.07–1.23)	1.10 (1.03–1.18)
Behavioural and emotional disorders^a^				
No maternal migraine	61 887	1.77	1 (Reference)	1 (Reference)
Maternal migraine	2193	2.44	1.28 (1.22–1.33)	1.23 (1.17–1.28)

a Behavioural and emotional disorders with onset usually occurring in childhood and adolescence; Model 1 adjusted for sex, birth year; Model 2 additionally adjusted for parity, maternal characteristic (age, education level, origin, cohabitation, cardiovascular diseases), paternal age, parental psychiatric disorders before the childbirth.

**Fig. 2.**
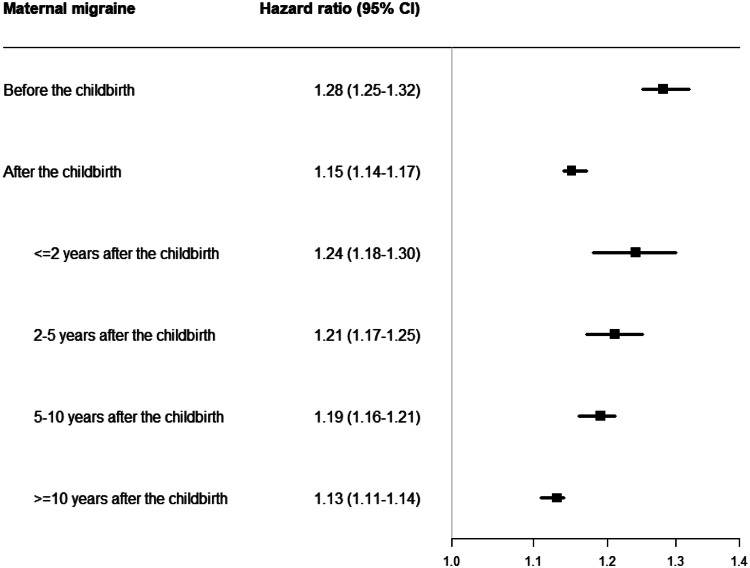
Hazard ratio and 95% CI for overall psychiatric disorders according to the timing of maternal migraine diagnosed.

We also observed associations between maternal migraine diagnosed after the childbirth and psychiatric disorders in offspring (HR, 1.15; 95% CI, 1.14–1.17). The overall HR is 1.24 (95% CI, 1.18–1.30) when the mother was diagnosed with migraine within 2 years after the childbirth, which is similar to the HR for prenatal exposure. But the HRs decreased over time in offspring of mothers diagnosed migraine within 2–5 years after the childbirth (HR, 1.21; 95% CI, 1.17–1.25), 5–10 years after the childbirth (HR, 1.19; 95% CI, 1.16–1.21) and more than 10 years after the childbirth (HR, 1.13; 95% CI, 1.11–1.14) (Fig. 2).

The highest overall risk of psychiatric disorders was observed in offspring of mothers with both migraine and comorbid psychiatric disroders (HR, 2.13; 95% CI, 1.99–2.28), comparing to offspring of mothers with migraine only (HR, 1.28; 95% CI, 1.24–1.32) (Table 4).

**Table 4.** Joint effect of maternal migraine and maternal psychiatric disorders before the childbirth on psychiatric disorders in offspring

Exposure	No. of offspring with psychiatric disorders	Rate per 1000	HR (95% CI)	HR (95% CI)
		person-years	Model 1	Model 2
No maternal migraine and psychiatric disorders	253 699	6.65	1 (Reference)	1 (Reference)
Maternal migraine only	4239	7.79	1.29 (1.25–1.33)	1.28 (1.24–1.32)
Maternal psychiatric disorders only	18 144	12.99	2.13 (2.10–2.16)	1.81 (1.78–1.84)
Joint effect of maternal migraine and psychiatric disorder	860	13.57	2.46 (2.30–2.63)	2.13 (1.99–2.28)

Model 1 adjusted for sex, birth year; Model 2 additionally adjusted for parity, maternal characteristic (age, education level, origin, cohabitation, cardiovascular diseases), paternal age, paternal psychiatric disorders before the childbirth.

When excluding offspring with adverse birth outcomes such as preterm birth, low birth weight and low Apgar score, the estimates remained unchanged (online Supplementary Table 3). Similar associations were observed in the analyses restricted to offspring born after 1991, 1996 or 2004, respectively, and when we use the multiple imputation for missing values of the covariates (online Supplementary Tables 4–7). We observed that the associations between maternal migraine and psychiatric disorders in offspring were similar for maternal migraine diagnosed prior to the index pregnancy and diagnosed during the index pregnancy (online Supplementary Table 8). Adverse birth outcomes probably accounted for only a very small proportion (0.10–1.95%) of the association between maternal migraine and risk of psychiatric disorders (online Supplementary Table 9).

## Discussion

In this large population-based cohort study, we found that maternal migraine was associated with an increased risk of any psychiatric disorders in offspring from childhood to early adulthood. Prenatal exposure to maternal migraine was associated with most specific psychiatric disorders, in particular, mood disorders, neurotic, stress-related and somatoform disorders and personality disorders. The highest risk of psychiatric disorders was observed in offspring of mothers with migraine and comorbid psychiatric disorders before childbirth.

### Interpretation of results and comparison with other studies

To our knowledge, the association between maternal migraine and risk of psychiatric disorders in offspring has only been investigated in a Finnish Prenatal study of Bipolar disorders (FIPS-B), in which a 1.5-fold risk of bipoloar disorders in offspring was reported (Sucksdorff *et al*., 2016). Consistently, we observed a similar magnitude of association between maternal migraine and mood disorders (including bipolar disorder) in offspring. However, in the Finnish study the information on psychiatric disorders was only available on bipolar disorders (Sucksdorff *et al*., 2016). To our knowledge, our study provided the novel evidence that offspring of mothers with migraine tended to be at an increased risk of any psychiatric disorders, persisting from childhood into early adulthood. Maternal migraine may lead to hypothalamic−pituitary−adrenal dysfunction (Galletti *et al*., 2009), fluctuating hormone and oxidative stress (Bernecker *et al*., 2011; Aggarwal *et al*., 2012; Neri *et al*., 2015), which can result in suboptimal intrauterine environment. Changes in the intrauterine environment could have a long-lasting effect on fetal brain development and, thus, increase the susceptibility to psychiatric disorders over the lifespan (Weinstock, 2005; O'Donnell and Meaney, 2017). Our study showed that maternal migraine diagnosed after the childbirth, especially diagnosed with migraine within 2 years after the childbirth, was associated with overall increased risk of psychiatric disorders in offspring. This is plausible because migraine may already be present for a period of time before the diagnosis (Wessman *et al*., 2007). As expected, the effect sizes associated postnatal exposure to maternal migraine decreased over time, supporting the programming effect of intrauterine environment on psychiatric disorders. On the other hand, we could not rule out the possibility that the observed association could be partly explained by the shared environmental risk factors (Sucksdorff *et al*., 2016).

Furthermore, we also observed that maternal migraine was associated with increased risks of most subtypes of psychiatric disorders in offspring, for example, mood disorders, neurotic, stress-related and somatoform disorders. Different types of psychiatric disorders may share common pathogenic mechanisms (Insel and Wang, 2010). For example, similar high level of transcriptomic overlap has been observed among mood disorders, personliaity disorders and pervasive developmental disorders (Gandal *et al*., 2018). Genome-wide analysis studies have also revealed substantial genetic overlap among different psychiatric disorders (Anttila *et al*., 2018; Martin *et al*., 2018). Moreover, shared neurocognitive endophenotypes, such as deficits in executive function, processing speed and working memory, have been described in most psychiatric disroders (McTeague *et al*., 2016).

We observed a two-fold increased risk of psychiatric disorders in offspring of mothers with both migraine and comorbid psychiatric disorders before the childbirth. The FIPS-B study also reported that the greatest risk was observed in offspring of parents with comorbid migraine and bipolar disorders (Sucksdorff *et al*., 2016). Even if the biological mechanism of coexisting maternal migraine and psychiatric disorders during pregnancy is unknown, genetic component can contribute to the highest incidence rate of psychiatric disorders in offspring (Wessman *et al*., 2007). Another possible explanation could be that mothers with co-morbid migraine and psychiatric disorders have a higher level of psychological stress (Minen *et al*., 2016). As a result, offspring may be exposed to more severe psychological stress *in utero*, which is associated with an increased risk of psychiatric disorders (Tegethoff *et al*., 2011; MacKinnon *et al*., 2018). Additionally, if the women diagnosed with migraine before the childbirth, she may exhibit increased circulating concentrations of inflammatory markers and could be aggravated by psychiatric disorders (Vanmolkot and De Hoon, 2007; Saunders *et al*., 2014); maternal inflammation has been proposed to play a role in the development of psychiatric disorders in offspring (Estes and McAllister, 2016). Further studies to elucidate the underlying biological pathways are warranted.

### Strengths and limitations of this study

Our study has several strengths. First, the present study is the first to examine the association of maternal migraine and psychiatric disorders in offspring using the entire population in a country (Denmark). The nature of register data minimises the possibility of recall and selection bias. Second, we had detailed information on parental psychiatric disorders, family socioeconomic status and maternal cardiovascular diseases. Adjustment for these potential confounders could allow us to disentangle the effect of maternal migraine on psychiatric disorders in offspring from the effects of these potential confounders. Third, we had a long follow-up with a maximum of age 39 years. Thus, we can investigate not only psychiatric disorders manifested in childhood or adolescence, but also psychiatric disorders such as schizophrenia spectrum disorders, mood disorders and adult personality disorders that are often diagnosed in adulthood (Kessler *et al*., 2007).

Our findings should be interpreted with caution due to several limitations. First, as in other observational studies there may still be potential residual confounding that could not be entirely eliminated (Fewell *et al*., 2007). For example, maternal pre-pregnancy body mass index, a risk factor for offspring psychiatric disorders (Mackay *et al*., 2017), might confound the observed association. However, additionally adjusting for pre-pregnancy BMI in women with available data did not change our results (shown in online Supplementary Table 6). Second, migraine was identified using a combination of hospitalisation registers and national prescription system, and the prevalence of migraine in our study is similar to another Danish population study (Le *et al*., 2012). However, the information on outpatient contact and medicine prescription were not available until 1995. This would lead to under diagnosis of maternal migraine before 1995, and some of them could be identified as postnatal migraine after 1995, which may underestimate the effect of prenatal exposure to migraine. Third, we could not rule out possible detection bias (Delgado-Rodriguez and Llorca, 2004). Children whose mothers with migraine are more likely to be in close contact with medical care than the unexposed children because of increased medical awareness, which might increase the opportunities to be diagnosed with psychiatric disorders. However, when investigating the specific psychiatric disorders with onset at different ages, varied risk estimates were observed. Thus, detection bias is unlikely to explain the association of maternal migraine with psychiatric disorders in offspring. Fourth, we chose not to adjust for perinatal factors such as preterm birth and low birth weight, as it has been shown that such adjustment may introduce bias (Hernández-Díaz *et al*., 2006). Furthermore, these neonatal characteristics could be potential mediators in the pathway from maternal migraine and psychiatric disorders in offspring (Mathewson *et al*., 2017; Skajaa *et al*., 2019). Nevertheless, our findings showed that the elevated risk of psychiatric disorders in offspring was not attenuated after excluding chidren of preterm birth, low birth weight or low Apgar score, as shown in the sensitivity analysis.

## Conclusion and policy implications

This study provides important information about offspring's mental well-being affected by maternal migraine using data from the Danish national registers. Given the high prevalence of migraine, especially among women at reproductive ages, our finding stands as strong evidence for concrete actions to be better management of women with migraine at reproductive ages and to screen mental health problems in their children.

## Data Availability

All data are stored at the secure platform of Denmark Statistics, which is the central authority on Danish Statistics with the mission to collect, compile and publish statistics on the Danish society. Due to restrictions related to Danish law and protecting patient privacy, the combined set of data as used in this study can only be made available through a trusted third party, Statistics Denmark (http://www.dst.dk/en/kontakt).

## Supplementary material

For supplementary material accompanying this paper visit http://dx.doi.org/10.1017/S2045796021000421. click here to view supplementary material

## Patient and public involvement

No patients were involved in setting the research question or the outcome measures, nor were they involved in developing plans for design or implementation of the study. No patients were asked to advise on interpretation or writing up of results. There are no plans to disseminate the results of the research to study patients or the relevant patient community.
