# Supplementary material for: Maternal migraine and the risk of psychiatric disorders in offspring: a population-based cohort study
Source: Epidemiol Psychiatr Sci. 2021 Jul 15;30:e55. doi: 10.1017/S2045796021000421 (PMC8318838; doi:10.1017/S2045796021000421)
Supplement: Supplementary file 1 [file S2045796021000421sup001.docx]

Supplement Table 1 The diagnostic classification of psychiatric disorders according to ICD-10 and ICD-8 diagnoses

| Diagnosis | | ICD-8 | ICD-10 | Start of  Follow up |
| --- | --- | --- | --- | --- |
| Overall Psychiatric disorders | 290-315 | | F00-F99 | Birth |
| Schizophrenia and related disorders | 295.x9, 296.89, 297.x9, 298.29-298.99, 299.04, 299.05, 299.09, 301.83 | | F20-F29 | 10 years old |
| Mood disorders | 296.x9 (excluding 296.89), 298.09, 298.19, 300.49, 301.19 | | F30-F39 | 10 years old |
| Neurotic, stress-related and somatoform disorders | 300.x9 (excluding 300.49), 305.x9, 305.68, 307.99 | | F40-F48 | 5 years old |
| Eating disorders | 305.60, 306.50, 306.58, 306.59 | | F50 | 6 years old |
| Specific personality disorders | 301.x9 (excluding 301.19), 301.80, 301.81, 301.82, 301.84 | | F60 | 15 years old |
| Mental retardation | 311.xx, 312.xx, 313.xx, 314.xx, 315.xx | | F70-F79 | Birth |
| Pervasive developmental disorders | 299.00, 299.01, 299.02, 299.03 | | F84 | Birth |
| Behavioral and emotional disorders with onset usually occurring in childhood and adolescence | 306.x9, 308.0x | | F90-F98 | Birth |

Supplement Table 2 Incidence rate and hazard ratios of overall psychiatric disorders in offspring according to maternal migraine status stratified by sex (n=2 069 785)

|  | No of offspring with | Rate per 1000 | Hazard ratio (95% CI) | Hazard ratio (95% CI) |
| --- | --- | --- | --- | --- |
|  | psychiatric disorders | person years | Model 1 | Model 2 |
| Male |  |  |  |  |
| No maternal migraine | 138 484 | 6.86 | 1 [Reference] | 1 [Reference] |
| Maternal migraine | 2927 | 9.52 | 1.28 (1.24 to 1.33) | 1.23 (1.19 to 1.28) |
| Female |  |  |  |  |
| No maternal migraine | 133 359 | 6.90 | 1 [Reference] | 1 [Reference] |
| Maternal migraine | 2172 | 7.24 | 1.38 (1.32 to 1.44) | 1.31 (1.25 to 1.36) |

Model 1 adjusted for birth year; Model 2 additionally adjusted for parity, maternal characteristic (age, education level, origin, cohabitation, cardiovascular diseases), paternal age, paternal migraine, parental psychiatric disorders before the childbirth

Supplement Table 3 Incidence rate and hazard ratios of overall psychiatric disorders in offspring excluding children with preterm birth, low birth weight and low Apgar score (n= 1 932 439)

|  | No of offspring with | Rate per 1000 | Hazard ratio (95% CI) | Hazard ratio (95% CI) |
| --- | --- | --- | --- | --- |
| Exposure | psychiatric disorders | person years | Model 1 | Model 2 |
| All offspring |  |  |  |  |
| No maternal migraine | 249 006 | 6.71 | 1 [Reference] | 1 [Reference] |
| Maternal migraine | 4568 | 8.10 | 1.32 (1.28 to 1.36) | 1.26 (1.23 to 1.30) |

Model 1 adjusted for birth year sex; Model 2 additionally adjusted for parity, maternal characteristic (age, education level, origin, cohabitation, cardiovascular diseases), paternal age, paternal migraine, parental psychiatric disorders before the childbirth

Supplement Table 4 Incidence rate and hazard ratios of overall psychiatric disorders in offspring excluding children born before 1991 (n=1 372 711)

|  | No. of offspring with | Rate per 1000 | Hazard ratio (95% CI) | Hazard ratio (95% CI) |
| --- | --- | --- | --- | --- |
|  | psychiatric disorders | person years | Model 1 | Model 2 |
| All offspring |  |  |  |  |
| No maternal migraine | 140 344 | 7.37 | 1 [Reference] | 1 [Reference] |
| Maternal migraine | 4396 | 8.22 | 1.28 (1.25 to 1.32) | 1.24 (1.20 to 1.28) |

Model 1 adjusted for birth year sex; Model 2 additionally adjusted for parity, maternal characteristic (age, education level, origin, cohabitation, cardiovascular diseases), maternal smoking status during pregnancy, paternal age, paternal migraine, parental psychiatric disorders before the childbirth

Supplement Table 5 Incidence rate and hazard ratios of overall psychiatric disorders in offspring excluding children born before 1996 (n= 1 051 058)

|  | No of offspring with | Rate per 1000 | Hazard ratio (95% CI) | Hazard ratio (95% CI) |
| --- | --- | --- | --- | --- |
|  | psychiatric disorders | person years | Model 1 | Model 2 |
| All offspring |  |  |  |  |
| No maternal migraine | 83 359 | 6.89 | 1 [Reference] | 1 [Reference] |
| Maternal migraine | 3936 | 7.92 | 1.26 (1.22 to 1.30) | 1.22 (1.18 to 1.26) |

Model 1 adjusted for birth year sex; Model 2 additionally adjusted for parity, maternal characteristic (age, education level, origin, cohabitation, cardiovascular diseases), maternal smoking status during pregnancy, paternal age, paternal migraine, parental psychiatric disorders before the childbirth

Supplement Table 6 Incidence rate and hazard ratios of overall psychiatric disorders in offspring excluding children born before 2004 (n=545 460)

|  | No of offspring with | Rate per 1000 | Hazard ratio (95% CI) | Hazard ratio (95% CI) |
| --- | --- | --- | --- | --- |
|  | psychiatric disorders | person years | Model 1 | Model 2 |
| All offspring |  |  |  |  |
| No maternal migraine | 22 928 | 5.39 | 1 [Reference] | 1 [Reference] |
| Maternal migraine | 1661 | 6.51 | 1.24 (1.18 to 1.30) | 1.22 (1.15 to 1.28) |

Model 1 adjusted for birth year sex; Model 2 additionally adjusted for parity, maternal characteristic (age, education level, origin, cohabitation, cardiovascular diseases), maternal smoking status during pregnancy, maternal pregestational body mass index paternal age, paternal migraine, parental psychiatric disorders before the childbirth

Supplement Table 7 Incidence rate and hazard ratios of overall psychiatric disorders in offspring including all children born during 1978 to 2012 using multiple imputation (n=2 069 785)

|  | No of offspring with | Rate per 1000 | Hazard ratio (95% CI) | Hazard ratio (95% CI) |
| --- | --- | --- | --- | --- |
|  | psychiatric disorders | person years | Model 1 | Model 2 |
| All offspring |  |  |  |  |
| No maternal migraine | 271 843 | 6.88 | 1 [Reference] | 1 [Reference] |
| Maternal migraine | 5099 | 8.40 | 1.32 (1.28 to 1.36) | 1.26 (1.23 to 1.30) |

Model 1 adjusted for birth year sex; Model 2 additionally adjusted for parity, maternal characteristic (age, education level, origin, cohabitation, cardiovascular diseases), paternal age, paternal migraine, parental psychiatric disorders before the childbirth

Supplement Table 8 The hazard ratios of overall psychiatric disorders in offspring according to maternal migraine (n=2 069 785)

|  | No. of Cases^*^ | HR (95% CI) Model 1 | HR (95% CI) Model 2 |
| --- | --- | --- | --- |
| No maternal migraine | 271 843 | 1 (Reference) | 1 (Reference) |
| Maternal migraine |  |  |  |
| Prior to pregnancy | 4831 | 1.32 (1.28-1.36) | 1.26 (1.22-1.30) |
| During the pregnancy | 265 | 1.30 (1.15-1.47) | 1.24 (1.10-1.40) |

*Number of offspring with psychiatric disorders; HR, hazard ratio; CI, confidence interval; Model 1 adjusted for sex and birth year; Model 2 additionally adjusted for parity, maternal characteristic (age, education level, origin, cohabitation, cardiovascular diseases), paternal age, paternal migraine and parental psychiatric disorders before the childbirth.

Supplement Table 9 Mediation analysis with adverse birth outcomes as potential mediators between maternal migraine and risk of psychiatric disorders in offspring (n=2 069 785)

| Variables | Odds Ratio (95% CI)^a^ |
| --- | --- |
| Low birth weight |  |
| Natural direct effect | 1.082 (1.047 to 1.118) |
| Natural indirect effect | 1.001 (1.001 to 1.002) |
| Total effect | 1.084 (1.049 to 1.120) |
| Proportion, %^b^ | 1.77 |
| Preterm birth |  |
| Natural direct effect | 1.087 (1.050 to 1.121) |
| Natural indirect effect | 1.002 (1.052 to 1.123) |
| Total effect | 1.089 (1.052 to 1.123) |
| Proportion, % | 1.95 |
| Low Apgar score |  |
| Natural direct effect | 1.086 (1.051 to 1.122) |
| Natural indirect effect | 1.000 (1.000 to 1.000) |
| Total effect | 1.086 (1.051 to 1.122) |
| Proportion, % | 0.10 |

^a^: Adjusted for sex, birth year, parity, maternal characteristic (age, education level, origin, cohabitation, cardiovascular diseases), paternal age, paternal migraine and parental psychiatric disorders before the childbirth; ^b^: Proportion mediated was calculated as log(natural indirect relationship)/log(total relationship)

Figure S1. The proportion of offspring born to mothers diagnosed with migraine by birth years
